# The Pericardial Body of *Ciona intestinalis* Contains Hemocytes and Degenerating Muscle Cells, But No Parasites

**DOI:** 10.1007/s11686-020-00323-x

**Published:** 2020-12-26

**Authors:** Lilly Rohlfs, Katja Müller, Thomas Stach

**Affiliations:** 1 Humboldt-Universität zu Berlin, Institut für Biologie, Molekulare Parasitologie, Berlin, Germany; 2 Humboldt-Universität zu Berlin, Institut für Biologie, Vergleichende Elektronenmikroskopie, Berlin, Germany

**Keywords:** Gregarines, Acidian, Tunicate, Marine invertebrate

## Abstract

**Purpose:**

A ventral heart positioned posterior to the branchial basket and equipped with a pericardium is homologous in tunicates and their sister group, the craniates, yet the tunicate model organism *Ciona intestinalis* features a pericardial body, a structure peculiar to few ascidian species. Here, we set out to distinguish between two competing hypotheses regarding the function of the pericardial body found in the literature: (H_1_) The pericardial body performs a role in the removal of dysfunctional myocardial cells, and (H_2_) it is a specialized niche of the immune system involved in defense against parasites.

**Methods:**

We used histological techniques, transmission electron microscopy, and PCR-based gene sequencing to investigate whether individual ascidians parasitized with apicomplexan protists show signs of infections within the pericardial body.

**Results:**

In individuals of *C. intestinalis* from the German North Sea infested with apicomplexan protists, the pericardial body contains numerous myocardial cells in various stages of degeneration while no remnants of parasitic cells could be identified.

**Conclusion:**

Thus, we conclude that H_2_—the pericardial body is a specialized niche of the immune system involved in defense against parasites—can be refuted. Rather, our observations support H_1_, the hypothesis that the pericardial body performs a role in the removal of dysfunctional myocardial cells.

**Supplementary Information:**

The online version contains supplementary material available at 10.1007/s11686-020-00323-x.

## Introduction

Generations of German-speaking morphologists have been introduced to the diversity of animal body plans with Kükenthal’s “Leitfaden für das zoologische Praktikum” as the principal textbook. The first edition came into print in 1898 [16] and its current 27th edition (under the name “Kükenthal—Zoologisches Praktikum”) with Volker Storch and Ullrich Welsch as authors is still the basis of many zoology courses at the university level [34]. One of the subjects of study in this book is the sea squirt *Ciona intestinalis*, now a well-known model organism for molecular developmental studies [20]. Starting with the 20th edition until today, in the chapter on *C. intestinalis*, aspiring morphologists stumble upon the didactically engaging and beautifully phrased sentence ‘Im Herz findet sich regelmäßig ein weißes “Bällchen” unbekannter Funktion’ (within the heart a little white ball of unknown function is regularly found—translation TS). Students, who too often assume that everything is known in morphology, are dependably inspired by this challenge. However, despite this prominent placement in an eminent textbook—what is the actual state of knowledge concerning this ‘little white ball’ in the primary literature?

In the zoological primary literature, the ‘little white ball’ is known as pericardial (or sometimes pericardic) body. The statement cited above, which locates the pericardial body in the heart, is not precise because the pericardial body is in fact situated within the pericardium. However, the main point, i.e., that the function remains unknown, is fundamentally correct, and primarily two competing hypotheses for its function are suggested. According to hypothesis 1 (H_1_), the pericardial body is the site of regulated degradation of senescent myocardial cells. The alternative hypothesis (H_2_) suggests that the pericardial body plays a crucial role in parasite removal as a central part of the immune system. H_1_ is based on the early light microscopical observation by Roule [27] that the pericardial body contained myocytes, and this observation was further supported in a study by Fernandez [12] and Millar [23]. However, because Fernandez astutely observed different types of hemocytes and erroneously thought the myocardial cells needed to transgress the epithelium of the pericardium, he nevertheless cast doubt on Roule’s original observations. Although ultrastructural observations confirmed the presence of degenerating myocytes and hemocytes in the pericardial body (e.g., Scippa and Izzo [31]), this research group later also observed apicomplexan, intracellular parasites in hemocytes mainly in the periphery of the pericardium of *C. intestinalis* species from the gulf of Naples [6, 32]. This observation led the authors suggest H_2_, i.e., that the pericardial body is a site of passive collection of these parasites. According to this hypothesis, the presence of myocardial cells in the pericardial body is a secondary result of their physical disruption caused by the constant passive movement of the pericardial body against the pericardial wall.

Of course, the two hypotheses are not mutually exclusive and the pericardial body might in fact serve a combination of both functions. In any case, while both hypotheses are in agreement with some observations, no study so far has shown the infestation with parasites simultaneously to an investigation of the ultrastructural morphology of the pericardial body. We argue; however, that this strategy would strengthen one of the hypotheses because.

If the myocyte–removal–hypothesis (H_1_) is true, then even in infected specimens, there should be ultrastructural evidence of degenerating muscle cells only in the pericardial body.

If, on the other hand, the parasitism hypothesis (H_2_) is true, then in infected specimens, there should be ultrastructural evidence of parasites in the pericardial body.

Thus, in order to distinguish between the two hypotheses, we studied several specimens of *Ciona intestinalis* for the presence of parasites using microscopic and molecular identification. We subsequently investigated the pericardial bodies of *C. intestinalis* specimens positively infected with gregarines using histology and transmission electron microscopy.

## Materials and Methods

### Animal Maintenance and Anesthetization

Living specimens of *Ciona intestinalis* (Linnaeus, 1767) were supplied in July 2018 by mail from the Biological Marine Station at the Alfred-Wegener-Institute on Helgoland. Animal size ranged from 5 to 12 cm when relaxed, i.e., animals were mature yet not fully grown. On Helgoland, only *C. intestinalis* is known to occur [33] and characteristics of the specimens used in the current study confirmed the species identity [3]. Animals were kept for up to 4 weeks in seawater tanks at 16–18 °C, ambient light and fed with approximately 1 ml Liquifry marine (Interpet Ltd., UK) per 10 l of seawater. Before further preparation, animals were anesthetized, by placing them individually in a 1 l glass beaker filled with micro-filtered (0.4-µm pore size) seawater. 2–3 crystals of menthol were added and the beaker containing animals, seawater, and menthol was placed in a refrigerator at 4 °C for 30 min.

### Electron Microscopy

Freshly dissected pericardial bodies were prefixed in 2.5% glutaraldehyde, 2% paraformaldehyde in 0.2 M phosphate buffer at pH7 and adjusted to an osmolarity of approximately 800 mosm with the addition of NaCl. Primary fixation was performed on ice and stopped after 45 min with three buffer rinses for 10, 15, and 20 min. For post-fixation, 1% OsO_4_ in 0.1 M phosphate buffer was used for 30 min on ice. The pericardial bodies were washed in double-distilled water, and were dehydrated in a graded ethanol series followed by propylene oxide and subsequent embedding in Araldite resin. The sections were cut using a diamond knife. Ultrathin sections (60 nm) were stained with uranyl acetate and lead citrate, and were analyzed using a Zeiss 900 transmission electron microscope. Images were recorded with a Wide-angle Dual Speed 2 k-CCD-Camera.

### Light Microscopy

Small individuals of *Ciona intestinalis* were fixed in Bouin’s fixative, an aqueous solution containing 8% formaldehyde, 5% acetic acid and 1% picric acid, for 48 h, dehydrated through an ethanol series, followed by paraffin-embedding in an automatic tissue processor (Shandon Hypercenter XP). Samples were subsequently sectioned at 10 µm thickness using a rotary microtome. The sections were mounted on glass slides and stained according to the Masson–Goldner–trichrome method.

Semi-thin sections  (1 µm) of araldite-embedded pericardial bodies (see section “Electron Microscopy”) were stained with toluidine blue.

Different tissue samples (branchial basket, intestinal tract, and body wall) of *C. intestinalis* were smeared directly on glass slides in a drop of seawater and examined for signs of parasite infections. These samples were observed either without fixation and without staining or after Giemsa staining.

Light microscopical examination was carried out on a Zeiss-Axioplan microscope equipped with a Canon Eos 700D.

### Sequence Analysis

#### Isolation of DNA, amplification by PCR, and sequencing

Genomic DNA was extracted from several tunicate tissues, specifically branchial basket, intestinal tract, body wall, and pericardial body. Tissues were cut into small pieces with a scalpel, followed by grinding with a pestle. Next, DNA was isolated with a QIAgen DNAeasy Kit, applying the animal tissue protocol after ATL-proteinase K digestion overnight. DNA content and quality were measured with a photospectrometer (NanoDrop). Polymerase chain reactions (PCR) were performed using a QIAGEN TopTaq Master Mix with 2–3 µl of genomic DNA as a template, and 1 µl of each primer (10 mM). In a nested PCR approach, to detect infections with marine gregarines, we first amplified SSU 18SrRNA with the generic primers F1 (GCGCTACCTGGTTGATCCTGCC) and R1 (GATCCTTCTGCAGGTTCACCTAC) for eukaryotes [18] and then amplified 1 µl of the first product as a template with primers F2 (GTDAATCGGCGTGTTCYACG) and R2 (GAATGCCCTCARCCGTTC) specific for lecudinid marine gregarines [29]. After visual analysis on agarose gels, PCR products of the expected size (128 bp) were sequenced at LGC genomics using both amplification primers. The identity of the gregarine sequences was verified to genus level via comparisons to reference sequences by NCBI BLAST.

## Results

**Fig. 1:**
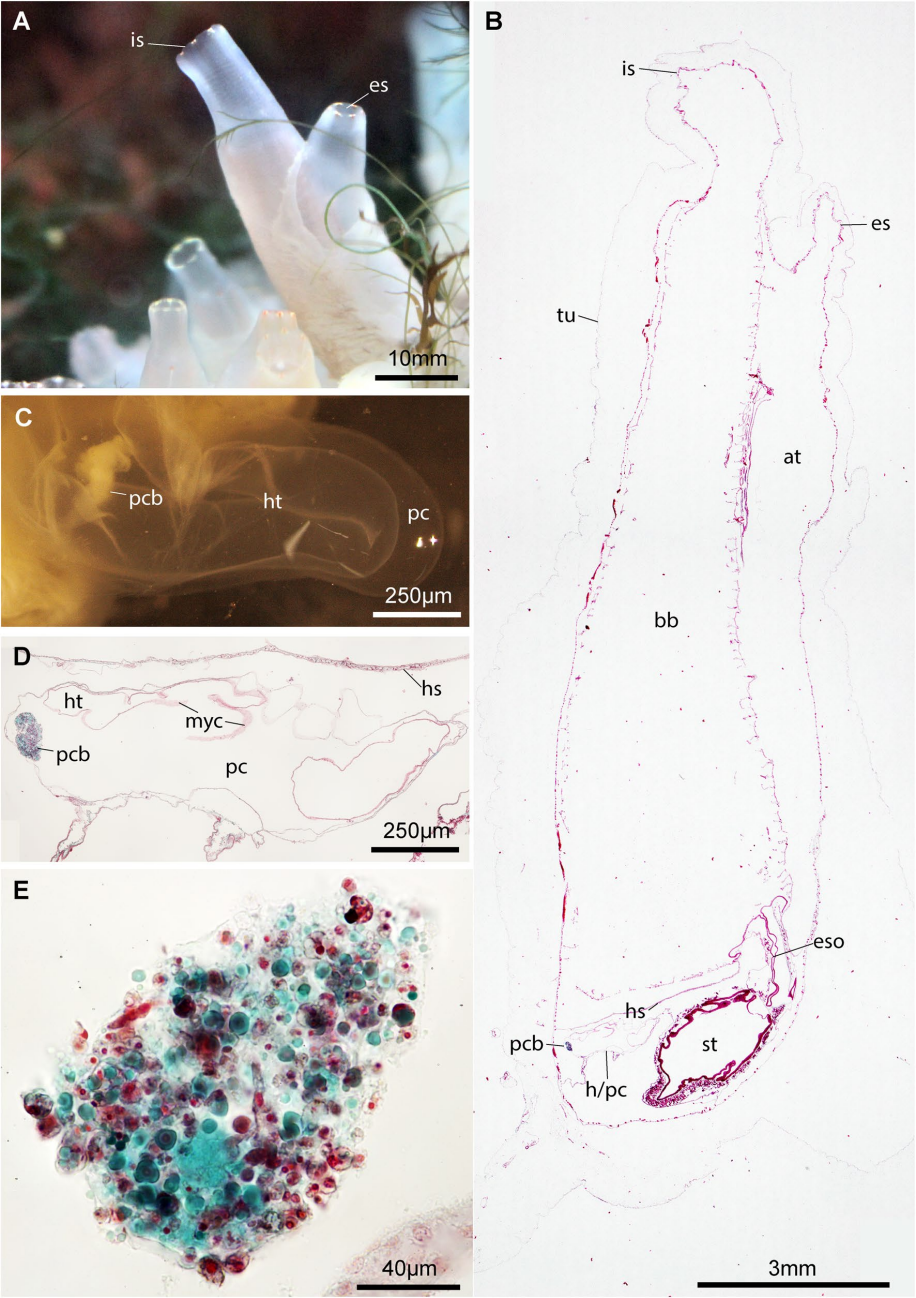
Pericardial body within the heart–pericard complex in the ascidian *Ciona intestinalis.* **a** Healthy, live individuals in feeding position show relaxed and opened siphons. Note colored eyespots around incurrent and excurrent siphons. **b** Light-micrograph of a parasagittal section showing inner anatomy. The pericardial body (pcb) is seen as a whitish structure of irregular shape. **c** Freshly dissected heart–pericard complex (h/pc). **d** Histological section of a h/pc sectioned along the longitudinal axis. **e** Light micrographic detail of histological section of a pcb. Note numerous round cells of different sizes, elongated cellular elements, and cellular fragments. *at *atrium, *bb* branchial basket, *es* excurrent siphon, *eso* esophagus, *h/pc* heart-pericard-complex, *hs* horizontal septum, *ht* heart, *is* incurrent siphon, *pcb* pericardial body, *st* stomach, *tu* tunic

In the pericardium of the ascidian model organism *Ciona intestinalis*, a white globular structure, the pericardial body, is regularly found (Fig. 1). Because two hypotheses regarding the function of the pericardial body exist, we combined morphological and molecular approaches in order to correlate parasite infection with an assessment of functional structure of the pericardial body.

### Gross examination

Eleven specimens of *Ciona intestinalis* were anaesthetized and their pericard–heart complexes dissected. A white pericardial body of irregular, often roughly oval shape and measuring approximately 100–250 µm, was present in each individual (Fig. 1c). For each individual, we dissected the pericardial bodies and either processed them for transmission electron microscopy (*n* = 4) or light microscopy (*n* = 5). In addition, we macerated tissue combined from the branchial basket, intestinal tract, and body wall of each individual (*n* = 11) for light-microscopic inspection and molecular analyses. Two pericardial bodies were also used for molecular analyses.

### Parasites

**Fig. 2:**
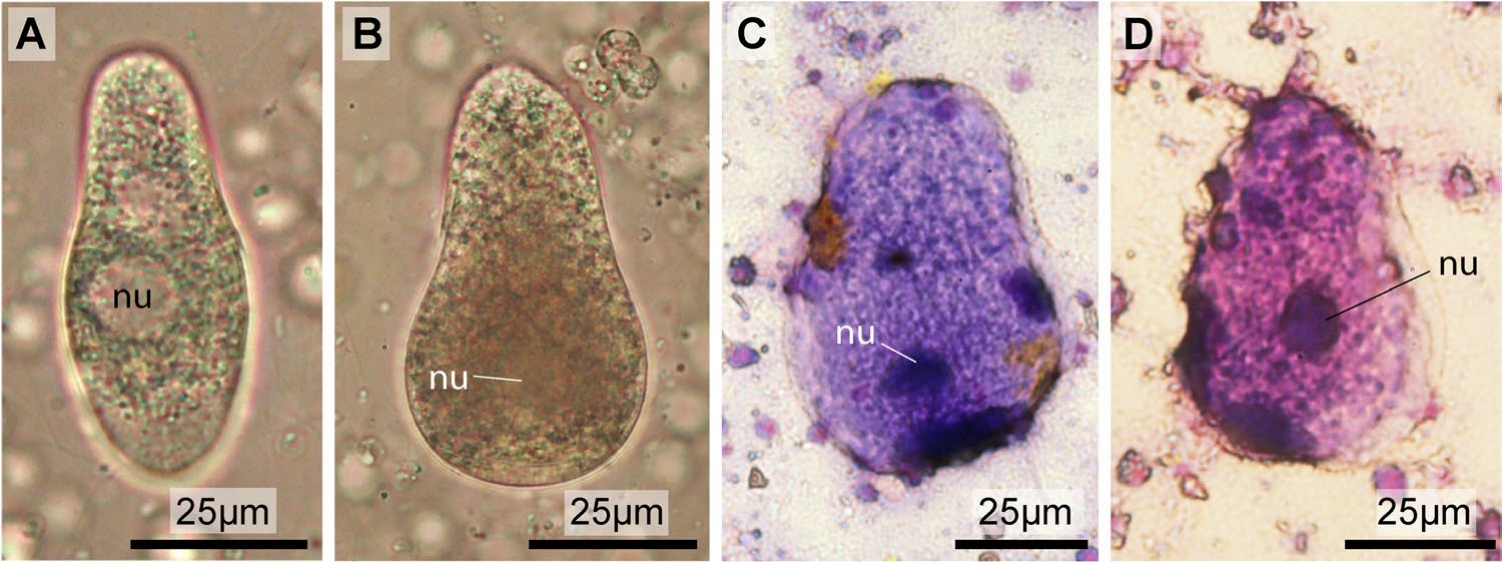
Light micrographs of gregarines in branchial basket tissue preparations. **a**, **b** living specimens. **c**, **d** Giemsa staining enhances visibility of the nucleus (nu)

The light microscopic inspection of the macerated tissues (branchial basket, intestinal tract, and body wall) showed that each individual was infected with parasitic gregarines (Fig. 2). The gregarines measured approximately 80 µm in length, were of pear shape to elongated shape, and featured a round prominent nucleus of approximately 8 µm diameter. Giemsa staining enhances visibility, but shrinks the gregarines to approximately 80% in linear dimensions. The distinction between a broader, blunter posterior end, and a narrower anterior tip characterized the gregarines as trophozoites.

Genomic DNA was isolated from the dissected tissues (branchial basket, intestinal tract, body wall, and pericardial body) and subjected to PCR amplification. Gregarine-specific primers [29], amplified s a 128 bp fragment of the 18SrDNA sequence from DNA isolated from branchial basket, intestinal tract, and body wall tissue, but not from pericardial body tissue. Of these fragments, 62–63 bp were high quality sequences in 5 of the 11 ascidians that were used in BLAST alignments (Figure S1, Supplementary Data). BLAST searches of these five sequences against the nucleotide database at NCBI resulted in 98% (2e−21–6e−21) sequence identity with only 1 consistent base mismatch and *Lankesteria ascidae 18SrRNA* (Gene Bank Sequence ID: JX187607 from position 765 to 826) as the only hit. BLAST searches with the sequences of the remaining 6 specimens yielded hits with two different species within the gregarine genus *Lankesteria*: *L. ascidiae* (*n* = 2, 2e−15–2e−17, 96% identity) and *L. cystodytes* (*n* = 4, 2e−14–2e−15, 96% identity). Both gregarine species have been isolated from ascidians from the Pacific Ocean, *L. ascidiae* from *C. intestinalis* [21, 24].

### Pericardial bodies

#### Histology

Light microscopic observation of paraffin-embedded pericardial bodies, stained with Masson–Goldner–trichromatic stains and sectioned at 10-µm thickness, showed that the pericardial bodies consisted of a conglomerate of cells and cellular fragments of various size and shapes (Fig. 1). Roundish to globular cells of approximately 10-µm diameter with dark brownish nuclei of around 5-µm diameter are numerous. The cytoplasm of these globular cells is predominantly greenish in coloration, indicating proteinaceous content at a lower pH. Elongated cells of 20 µm length and a diameter of 5–8 µm are also regularly encountered. The cytoplasm of these elongated cells is reddish in coloration, and the nucleus is darker stained. There are also large aggregations of cellular material, sometimes with several nuclei. Besides the larger globular cells, globular cells of merely 5 µm in diameter are numerous. The coloration of the globular cells is varied; the cytoplasm is mostly greenish, but sometimes transparent and numerous vesicles of different colors are present.

**Fig. 3:**
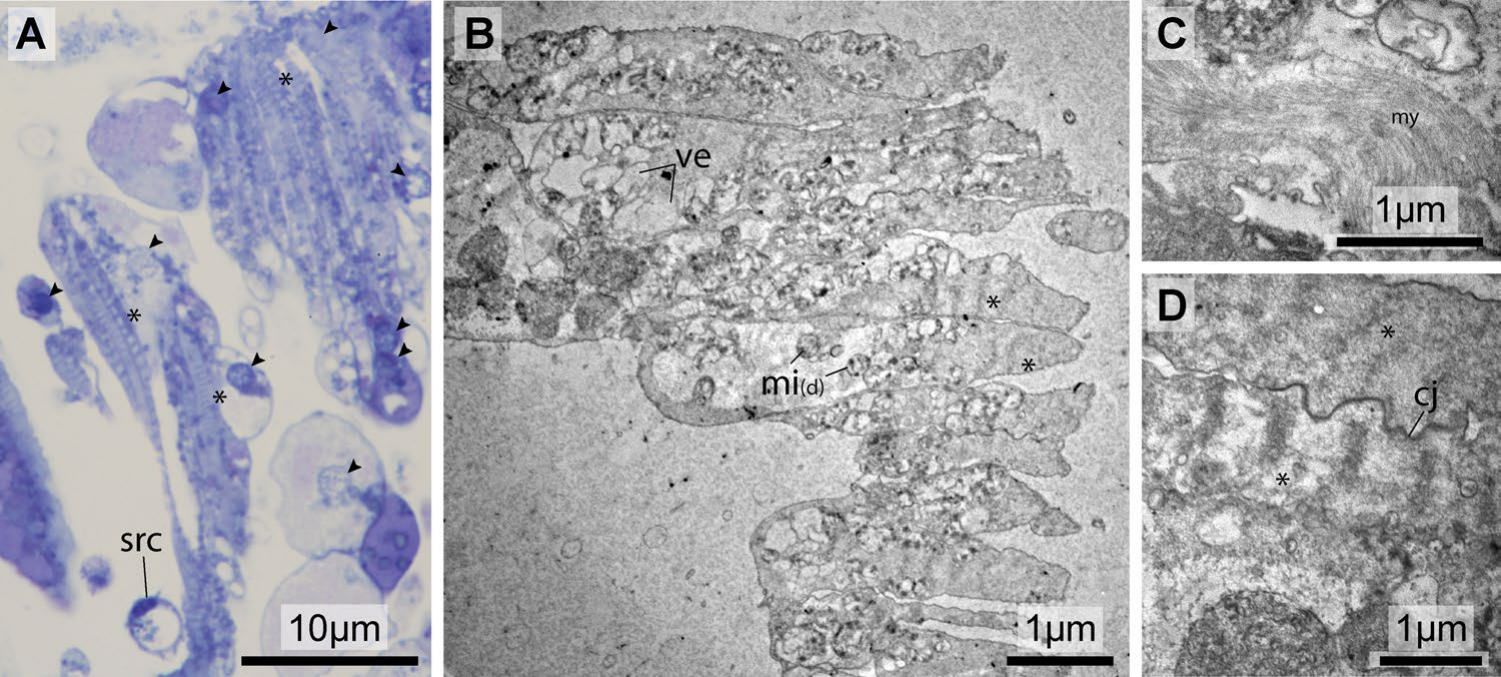
Histological and ultrastructural details of the pericardial body. **a** Semithin section stained with toluidine blue showing hemocytes and myocardial cells. **b** Transmission electron micrograph of partly degenerated myocardial cells. **c** Myofilaments in degenerating myocardial cell. **d** Detail of sarcomere in a degenerating myocardial cell. *cj* cell junction, *mi*_*(d)*_ degenerating mitochondria, *my* ofilaments, *src* signet ring cell, *ve* –vesicles; arrowheads: nuclei, asterisks: areas with sarcomeres

**Fig. 4:**
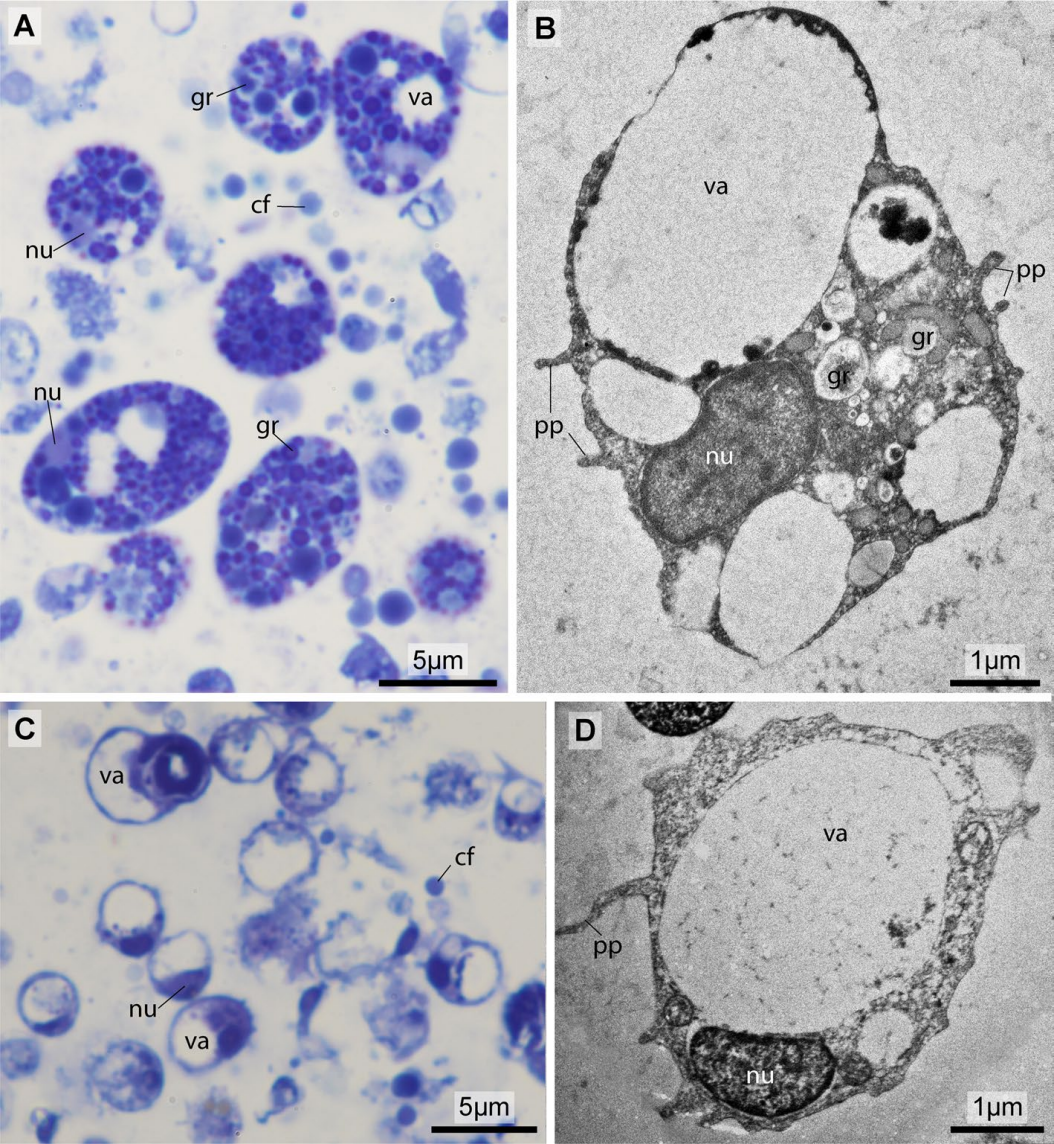
Histological and ultrastructural details of hemocytes within the pericardial body. **a**, **c** Semithin section stained with toluidine blue. **b**, **d** Transmission electron micrographs. **a**, **b:** Granular amoebocytes. **c**, **d:** Signet ring cells. *cf* cell fragment, *gr* granulum, *nu* nucleus, pp pseudopodium, *va* vacuole

In semithin sections (0.7 µm) stained with toluidine blue, the elongated cells often feature a repeated banding pattern of dark and light stripes (Fig. 3a), typical of sarcomeres of myocytes. In these histological preparations, several of such myocytes, with more or less distinct bands of sarcomeres are regularly found clumped together. In some of these myocytes, nuclei show the normal pattern of homogenous light blue euchromatin and darker spots of heterochromatin, whereas some nuclei appear unusually light with granular specks. In the latter cells, the cytoplasm shows vacuolated areas and degenerating organelles. Two kinds of other cells can be discerned: A conspicuous cell type with a single larger vacuole. This vacuole limits the cytoplasm to a thin margin between vacuole and cell membrane giving the cell a ring like appearance in cross section. With the nucleus protruding from this thin area, the appearance is more accurately described as that of a signet ring (Figs. 3a, 4c). The other cell type usually possesses several smaller vacuoles that appear empty and numerous dark granules that are evenly stained (Fig. 4a).

### Ultrastructure

#### Muscle Cells

Transmission electron microscopy revealed that the myocytes were at different stages of degeneration. The typical banding pattern of the sarcomeres was still discernible due to the repeated occurrence of darker *z* lines. The length of a sarcomere, however, differed between cells, ranging from 0.2 µm to more than 1 µm, with the *z* lines in various stages of disintegration, becoming successively more dilated (Fig. 3b). In some of the degenerating myocytes, myosin fibers could be identified, although the arrangement in sarcomeres was no longer recognizable (Fig. 2c). Dilatation and confluence of elements lead to internal fragmentation. Lysosomes, areas with vesicularized membrane-bound irregular bodies, and degenerating organelles, which are mostly mitochondria, if discernible, predominate the cytoplasm of these degenerating myocytes.

#### Hemocytes

Between the degenerating myocytes, two other cell types are seen: granular amebocytes and signet ring cells. Granular amebocytes (Fig. 4b) possess several electron lucent vacuoles with little precipitation as content and numerous granules with more electron dense content. In these granular amebocytes the periphery of the cells is extended into pseudopods. Electron microscopy clearly illustrates the morphology of the signet ring cells (Fig. 4d). These signet ring cells show a single larger vacuole, a narrow rim of cytoplasm, and a protruding nuclear region, and adds the occurrence of pseudopods in this cell type as well (Fig. 4d).

No signs of parasites could be discovered in histological preparations and were also not found in the ultrastructural preparations (see Figs. 3, 4).

## Discussion

According to phylogenomic analyses, tunicates comprise the most likely sister group to vertebrates within chordates [10, 15], with typical chordate characters such as a dorsal brain, a larval postanal tail in most species, and a branchial basket. Like all chordates, tunicates possess a heart surrounded by a pericardium on the ventral side, immediately posterior to the branchial basket [2, 14]. Based on these and additional developmental similarities, the tunicate heart is considered a homolog of the vertebrate heart [1, 8, 30]. Most tunicates are sessile ascidians and some ascidian species possess a pericardial body, a little white ball, found within the pericardium. It is regularly found in *Ciona* spp., but has also been reported occasionally in other species in the family Ascidiaceae (e.g., Fernandez 1907; Kalk [14]. Regarding the functional significance of this pericardial body two main hypotheses have been suggested: hypothesis 1 (H_1_) suggests that the pericardial body is a site of degradation of discarded myocardial cells (e.g., Roule [2], Millar [23], Burighel and Cloney [27]), whereas according to hypothesis (H_2_) the pericardial body functions in parasite removal [31, 32].

In principal, these hypotheses should be discernible by simultaneously investigating the infection status of specimens and the ultrastructure of the pericardial body in infested individuals: we argue that in infected specimens, there should be ultrastructural evidence of degenerating muscle cells in the pericardial body, if H_1_ is true. If, on the other hand, H_2_ is correct, then in infected specimens, there should be ultrastructural evidence of parasites in the pericardial body.

We investigated a total of 11 specimens and found that every individual was infested with apicomplexan parasites, a prevalence of 100%. Apicomplexan parasites have been reported from different anatomical compartments in their respective hosts, e.g., from the intestinal tract [5, 26], coelomic cavities, or the hemocoel [11, 28]. Based on the sequence comparison of a partial region of the 18S rDNA-gene, we could identify the parasites as species within the intestinal parasite gregarine genus *Lankesteria*, most probably *L. ascidiae* nested within Apicomplexa (e.g., Leander [17], Morrison [25]). Despite the relative shortness of the partial 18S rRNA-gene we amplified, we are confident that the gregarines belong to the genus *Lankesteria*, while the species attribution remains more capricious. We analyzed the ultrastructure of the pericardial bodies of individuals, all of which were infected with *Lankesteria* spp., and did not find evidence of parasites within the pericardial bodies. Therefore, we conclude that H_2_ is not supported by this study, and we strengthen H_1_, the hypothesis that the pericardial body is a site of removal of accumulating, degenerating apoptotic myocardial cells within the pericardium. Of course, this does not preclude the possibility that other gregarine parasites infiltrate other body compartments, such as the hemocoel [11], coelomic cavities in general [19], or specifically the pericardium as was observed by Scippa et al. [32] and Cianco et al. [6]. This would of course be the case for the intracellular parasites, such as *Cardiosporidium cionae*, observed by these authors within hemocytes in the pericardium [6, 32]. We could not find histological or ultrastructural evidence of such an intracellular infestation. Moreover, our attempts to identify an infection with *C. cionae* by amplifying a partial sequence of the 18srRNA gene using a nested approach by first amplifying SSU 18SrRNA with the generic primers F1 (GCGCTACCTGGTTGATCCTGCC) and R1 (GATCCTTCTGCAGGTTCACCTAC) for eukaryotes [18] and then using the product as a template with primers designed to specifically amplify *C. cionae* 18SrRNA (GenBank accession: EU052685) could not pinpoint such an infestation.

Similar to the findings reported by Scippa and Izzo [31], we observed hemocytes in the pericardium. We identified granular amebocytes and signet-ring cells in the present study, and these hemocytes possess pseudopodial extensions, are capable of phagocytosis (e.g., de Leo [2, Burighel and Cloney [9]), and engulf the apoptotic cardiomyocytes and their remnants. Hemocyte-mediated phagocytosis is the primary innate immune defense of ascidians [7], and these can serve roles in parasite removal [4, 9].

For the electron and light microscopic investigation, we intentionally selected smaller, and thus younger individuals. In these smaller and younger specimens, parasites might not have penetrated into the pericardial coelomic cavity in high enough numbers to be detected in light microscopic, histological, or electron microscopic preparations. This line of reasoning, could reconcile the two hypotheses H_1_ and H_2_: the primary function of the pericardial body is seen in the removal of discarded, apoptotic myocardial cells (H_1_), which is facilitated by hemocytes. These hemocytes can also serve as a line of defense, in case parasites infect the coelomic cavity of the pericard as has been shown by Scippa et al. [32]. In conclusion, we strengthen the case in favor of hypothesis H_1_, i.e., that the main and primary function of the pericardial body lies in the removal of degenerating myocytes, but the phagocytic hemocytes involved in this process might also take up parasites in the pericardium. In any case, despite its didactic value, we suggest that in the next edition of the textbook “Zoologisches Praktikum” the words “unbekannter Funktion” (= of unknown function) are omitted when referring to the pericardial body.

Incidentally, the observation of hemocytes in the pericardium indicates that the cells are capable of leaving the blood stream and migrating through epithelially organized pericardial cells to reach the sites where they fulfill their functional roles. This cellular behavior is similar to leucocytes in vertebrates, where this behavior is termed diapedesis (e.g., Marchesi and Florey [13], Filippi [22]. The confirmation of apoptotic cardiomyocytes in the pericardial body also implies a general turnover of these muscle cells in the heart of *C. intestinalis*. While molecular details of such a process are known in vertebrates (e.g., Takemura *et al.* [36], Vujic et al. [37]) and invertebrate model organisms (e.g., Zhu et al. [38]), hardly anything seems to be known of this process in marine invertebrates. The report of differentially proliferating and apoptotic heart muscle cells in two bivalve species [35], however, indicates that the tightly regulated turnover of heart muscle cells is a widespread phenomenon. The heart of *C. intestinalis*; therefore, is a promising model to elucidate this process also from an evolutionary and molecular perspective.

## Supplementary Information

Below is the link to the electronic supplementary material.

Supplementary Figure S1. A: Agarose gel electrophoresis of PCR-amplified DNA segments. Left: amplified SSU 18SrRNA gene using eukaryote primers F1 and R1 (see Materials and Methods for details). Right: amplified partial sequence of SSU 18SrRNA gene using 1µl of the first product as a template with primers F2 and R2 (see Materials and Methods for details) specific for lecudinid marine gregarines. B: The results of BLAST-searches on GenBank with two representative sequences of partial 18SrRNA gene sequences amplified from tissue isolated from two different specimens of the ascidian Ciona intestinalis (see Materials and Methods for details). C: Colored alignment of the two representative sequences of partial 18SrRNA gene sequences shown in B. First line shows the reference sequence of the gregarine Lankesteria ascidiae (Genbank accession number JX187607.1). The single nucleotide difference is highlighted by the exclamation mark
